# Revealing the Corrosion Resistance Mechanism of Plain Carbon Steel Micro-Alloyed by La in Simulated Industrial Atmosphere

**DOI:** 10.3390/ma17184467

**Published:** 2024-09-11

**Authors:** Sha Sha, Feng Yang, Jianzhong He, Zhi Liu, Tianle Fu, Bing Wang, Xiaoping Chen, Shujun Jia, Qingyou Liu

**Affiliations:** 1 Central Iron and Steel Research Institute Limited Company, Beijing 100081, China; shashasayyes@163.com (S.S.); liuzhismsc@163.com (Z.L.); 18574437760@163.com (T.F.); chenxiaoping@cisri.com.cn (X.C.); jiashujun@cisri.com.cn (S.J.); liuqingyou@cisri.com.cn (Q.L.); 2 College of New Energy and Materials, China University of Petroleum (Beijing), Beijing 102200, China; 3 Inner Mongolia Key Laboratory of Rare Earth Steel Production Research and Development, Inner Mongolia Baotou Steel Union Co., Ltd., Baotou 014010, China; peak1978yang@126.com (F.Y.); hejianzhong666@foxmail.com (J.H.); 4 Technical Center of Inner Mongolia Baotou Steel Union Co., Ltd., Baotou 014010, China

**Keywords:** plain carbon steel, rare earth element of La, industrial atmosphere, corrosion resistance mechanism, corrosion inhibition efficiency

## Abstract

Plain carbon steel is the most widely applied steel in current engineering construction. With the increased application property needs, the service life of plain carbon steel has been severely tested. As one of the most destructive failure modes, corrosion resistance of carbon steel has attracted wide attention. Rare earth La, as the microalloying element, was employed in plain carbon steel, Q355, to improve its corrosion resistance. As the content of La increased, the microstructure was refined. The fraction of pearlite decreased, while the content of acicular increased. Within the La addition of 230 ppm, the tensile strength and impact energy were jointly improved. Furthermore, the microalloying element of La modified the inclusion types and refined the inclusion size. The modified microstructure and inclusions by La co-improved the corrosion resistance. The formula of effective La content was proposed to estimate the effect of La on corrosion. As the effective content of La increased, the relative corrosion rate decreased. La^3+^ promoted the protective rust layer to increase corrosion resistance.

## 1. Introduction

Light weight advanced steel materials possess superior comprehensive mechanical properties, such as high strength, excellent ductility, and good low-temperature toughness [1,2]. With the rapid development of design theory, advanced steel materials have been widely used in engineering areas [3]. Q355, as a plain carbon steel, plays important roles in social constructions, facing serious failure risks [4,5]. Among these risks, corrosion degrades materials, resulting in significant economic losses, safety risks, and environmental concerns that have a serious impact on the global industry [6]. In order to increase the corrosion resistance, the corrosion-resistant steel was developed in the mid-20th century [7]. Corrosion-resistant steels, with total addition of Cu, P, Cr, Ni, Si and Mn no more than 3–5 wt.%, are low-alloyed steels [8,9]. The corrosion resistance of these corrosion-resistant steels is 2 to 8 times that of plain carbon steel [10]. However, the high cost of these steels limits their application in the field of general atmospheric and marine corrosion resistance. Therefore, reducing the cost of corrosion-resistant steel and expanding its application range are crucial research areas in the steel industry.

To decrease the use-cost and propel further industrial application, there are developed ways: coating [11], cheaper anti-corrosion alloying elements [12], and other materials. In recent decades, the role of rare earth (RE) elements in increasing the corrosion resistance in steel has gained more and more attention [12,13,14]. Studies show that the main mechanism for RE element optimizing the corrosion resistance is from the point of view of improving inclusions [13,15]. Inclusion in steel is considered dangerous, but it is an indispensable product in the process of the deoxidation and desulfurization of steel. It is widely accepted that pitting corrosion is the main negative influence mechanism caused by inclusions on corrosion resistance [16,17]. It is well known that sulfide and oxide inclusions usually have a different corrosion potential with matrix, thereby leading to the initial nucleation nuclei of pitting corrosion in plain carbon steel. The similar negative effect of TiN on the corrosion resistance was also reported [18]. As the sizes of inclusions decrease, the pit nuclei are hard to transform into steadily growing pits. Hence, it is necessary to design and control the inclusions in steel to optimize the corrosion resistance of steel.

RE elements displaying strong affinity with sulfur and oxygen were employed to improve inclusions during steelmaking [19]. Therefore, microalloying RE elements could play important roles in purifying molten steel and modifying inclusions [20]. Liu, et al. found that La and Ce are strong inhibitors to decrease the pitting tendency in a marine environment of plain carbon Q460NH weathering steel [13]. The potential of these modified inclusions is about 120 mV lower than the matrix, triggering the pitting from the matrix near the inclusions to the inclusion interior. Besides, the corrosion resistance mechanisms of rare earth elements also connect with the interaction between RE and other alloy elements. However, the systematic studies on the existence of forms of RE in steel are completely ignored, as well as the influence of the corrosion inhibition of RE elements on the corrosion resistance.

In the present work, the different contents of La were designed in Q355D plain steel and the effect of La on the weathering resistance under the simulated industrial atmospheric corrosion environment was studied using physicochemical phase analysis, electrochemical testing, scanning electron microscopy (SEM) and X-ray diffraction (XRD).

## 2. Materials and Methods

### 2.1. Sample Preparation

The chemical composition of La-modified Q355 plain carbon steel is displayed in Table 1, where four gradients of La were designed. After 50 kg vacuum induction furnace melting and casting, the Fe alloy was forged into a 50 mm × 90 mm square billet. Subsequently, a series of rolling passes reduced the billet thickness to a 7 mm plate. The rolling process was as follows: homogenization preservation at 1200 °C for 1 h, three passes from 1200 °C to 1000 °C, and three passes from 1000 °C to 860 °C. After water cooling, the plate was coiled at 550 °C.

### 2.2. Tensile Property Testes and Microstructure Characterizations

Round tensile samples with a gauge length of 18 mm, diameter of 3 mm, and total length of 43 mm (along the rolling direction) were prepared. The GNT100 electronic tensile testing machine (NCS, Beijing, China) was used to estimate the tensile properties at a strain rate of 1 mm/min. Charpy V-notch samples with 10 mm × 10 mm × 55 mm were also prepared along the rolling direction and performed at the JBN-300B impact machine (Jinan Hengda Huifeng Test Instrument Co., Ltd., Jinan, China) at ambient temperature to estimate the impact energy. Tensile properties depended on two values, while impact energy relied on three samples.

The samples for observing the microstructure were prepared from the coiled steel along the rolling direction. Before observation, the samples were subjected to the standard metallographic procedure: mechanically grinding by SiC sandpapers from 320#, 600#, 1000#, and 2000# sequence, poshing using the diamond plaster of about 1.5 μm, and erosion in a 4% nital alcohol solution. The metallographic sample was observed in Axio Observer (Zeiss series). Further, the inclusion was analyzed by FEI Quanta FEG 650 SEM equipped with mapping EDS.

### 2.3. Alternate Immersion Tests

A series of alternate immersion tests were conducted to study the accelerated corrosion behavior in 0.01 mol/L NaHSO_3_ solution. The NaHSO_3_ solution is employed to simulate the industrial atmospheric corrosion environment and 500 mL of deionized water was added to each tank every day. The samples were processed into 60 mm × 40 mm × 3 mm corrosion samples according to the standard of TB/T 2375-1993 [21], and the corrosion cycle of the tested steel was 72 h. RE steels and plain carbon steel were hung in each tank, and the rust layer samples of 15 mm × 15 mm × 3 mm were hung at the same time, and there were 4 parallel samples for each steel. The corrosion weight loss method was used to calculate the corrosion rate. First, 500 mL 36.5% hydrochloric acid, 500 mL deionized water, 10 g hexamethylenetetramine and 3 g benzotriazole pickling were used to remove the rust layer on the surface. The pickling pattern was immediately put into an alcohol solution to prevent rust and then dried. The rust removal pattern is weighed with a weighing instrument with an accuracy of 0.0001 g. The corrosion rate was estimated by the equation:

(1) $$W=\frac{W_{t}\times 10^{6}}{2\left(a\times b+a\times c+b\times c\right)\times t}$$

(2) $$W_{t}=w_{0}-w_{t}$$

where ***W_t_*** was the change in the weight of the specimen before and after the test, unit: g; ***w*_0_** was the initial weight of the sample, unit: g; ***w_t_*** was the weight of the sample after scaling under the corrosion time of ***t***, unit: g; ***a***, ***b*** and ***c*** are the length, width, and height of the sample respectively, unit: mm. The relative corrosion rate, ***W*_R_**, was estimated as follows:

(3) $$W_{R}=\frac{W_{t-\mathrm{La}}}{W_{t-0}}\times 100\%$$

where ***W_t_***_−__La_ and ***W_t_*_−__0_** were the corrosion rate of La-bearing steel and plain carbon steel, respectively.

### 2.4. Electrochemical Tests

The electrochemistry workstation (Princeton, NJ, USA), equipped with a traditional three-electrode cell, was used to measure the polarization curve and electrochemical impedance spectroscopy (EIS). The tested steel with and without a rust layer was the working electrode (1 cm^2^), the platinum (Pt) electrode was the auxiliary electrode and the saturated calomel electrode (SCE) was the reference electrode. When the electrochemical system is stabilized, the frequency range of EIS is 10^−2^~10^5^ Hz, and the interference voltage amplitude is 10 mV. The scanning rate is 0.5 mV/s and the scanning range is ±500 mV for polarization curves. At room temperature, the test medium solution was 0.01 mol/L NaHSO_3_ solution with different mass concentrations of LaCl_3_·7H_2_O. A period of 30 min was needed to obtain open circuit potential. Subsequently, the electrochemical impedance spectroscopy (EIS) was carried out at the open circuit potential using AC signals of 10 mV amplitude (peak-to-peak sinusoidal wave) between 100 kHz and 10 mHz, with 10 points per decade. The EIS data was post-processed by ZSimpWin software 3.60 with a suitable equivalent circuit (EC).

### 2.5. Corrosion Inhibition Tests

The pure La RE specimen was placed in 0.01 mol/L NaHSO_3_ (pH = 3.2) solution, and it was found that the pure La RE specimen could dissolve in acidic NaHSO_3_ solution, releasing La^3+^ ions. The plain carbon steel was corroded into 0.01 mol/L NaHSO_3_, and a mixed solution of 0.01 mol/L NaHSO_3_ and 0.75 g/L La. The surface morphology of the specimen was observed.

### 2.6. Rust Analysis

The corrosion products were removed from the sample surface and then ground into powder. The corrosion products were characterized using D8 advanced XRD (Bruker AXS, Karlsruhe, Germany). The rust morphology of the rust layer was characterized by FEI (Hillsboro, OR, USA) Quanta FEG 650 SEM equipped with mapping EDS. The rust chemical distributions were tested by electron probe microanalysis (EPMA) in the JXA8230 device (JEOL, Tokyo, Japan).

## 3. Results

### 3.1. Mechanical Properties and Microstructure Characterization

**Figure 1:**
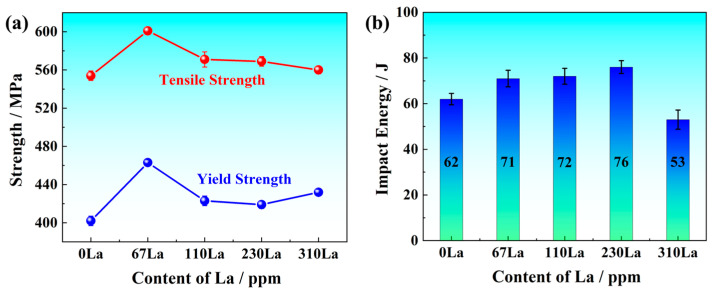
Mechanical properties: (**a**) Tensile strength and yield strength; (**b**) Impact energies at ambient temperature.

The tensile properties and impact toughness are displayed in Figure 1. The yield strength was above 400 MPa, while the tensile strength was higher than 600 MPa (Figure 1a). When the La content of 67 ppm was added, the tensile strength and yield strength increased by 47 and 61 MPa, respectively. As the content of La increased from 67 ppm to 310 ppm, the tensile strength decreased from 601 MPa to 560 MPa, while the yield strength decreased from 463 MPa to 419 MPa and then increased to 432 MPa.

The impact toughness was 62 J of 0La steel. Micro-alloyed by La, the impact energies increased to 71, 72 and 76 J. As the content of La increased to 310 ppm, the impact energy decreased to 53, indicating that the impact toughness strongly depended on the content of La.

**Figure 2:**
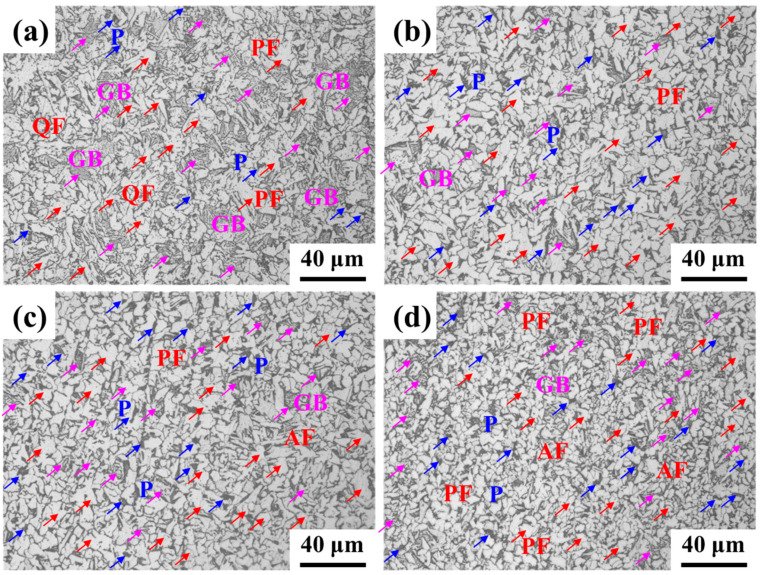
Microstructure of La-bearing steel: (**a**) 67La; (**b**) 110La; (**c**) 230La; (**d**) 310La.

The microstructure of La-bearing steel is shown in Figure 2. As the content of La increased, the microstructure was refined, which is consistent with the observed result by Torkamani, et al. [22]. It was a mixed microstructure of pearlite, polygonal ferrite, granular bainite and quasi-polygonal ferrite for 67La steel (Figure 2a). As the content of the addition content of La increased, the fraction of granular bainite significantly decreased. It was noted that the content of pearlite increased, and the polygonal ferrite and granular bainite were refined to 110La steel. The pearlite is mainly located at the ferrite grain boundaries (Figure 2b). With an increase of La content to 230 ppm, the microstructure was further refined (Figure 2c). Besides, the acicular ferrite appeared. The acicular ferrite both increased strength and guaranteed excellent low-temperature toughness [23]. As the content of La increased to 310 ppm, the fraction of pearlite significantly decreased, and the fraction of acicular ferrite increased (Figure 2d). 

The microstructure of La-bearing steel is shown in Figure 2. As the content of La increased, the microstructure was refined, which is consistent with the observed result by Torkamani, et al. [22]. It was a mixed microstructure of pearlite (P), polygonal ferrite (PF), granular bainite (GB), and quasi-polygonal ferrite for 67La steel (Figure 2a). As the addition content of La increased, the fraction of granular bainite significantly decreased. It was noted that the content of pearlite increased, and the polygonal ferrite and granular bainite were refined in 110La steel. The pearlite is mainly located at the ferrite grain boundaries (Figure 2b). With an increase of La content to 230 ppm, the microstructure was further refined (Figure 2c). Additionally, acicular ferrite (AF) appeared, which both increased strength and guaranteed excellent low-temperature toughness [23]. As the content of La increased to 310 ppm, the fraction of pearlite significantly decreased, and the fraction of acicular ferrite increased (Figure 2d).

### 3.2. Inclusion Analysis

The inclusions in the La-bearing steel were classified according to GB/T10561-2005 [24]. After the addition of La, the number of plate-like A-type sulfides in the microstructure is reduced. This indicates that the inclusions can be modified into D-type, small-size, and approximately circular inclusions by adding a trace amount of 110 ppm La in the liquid steel. Plain carbon steel with high La content is mainly composed of B-type and D-type inclusions. In addition, under the La addition of 310 ppm, A-type inclusions have been eliminated and the number of D-type oxide inclusions significantly increased. The addition of 310 ppm La significantly improved the characteristics of inclusions, effectively reducing the number of inclusions, leading to the original inclusions into smaller sizes and a more uniform distribution of inclusions.

**Figure 3:**
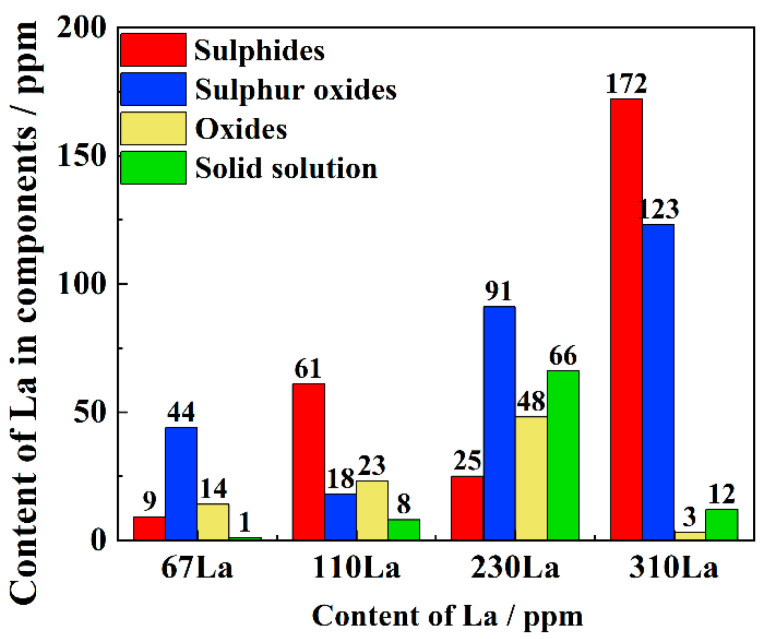
Physicochemical phase analysis result showing the content of La in three-type inclusions and the solution state.

The existence form of La was estimated by the physicochemical phase analysis. After removing the steel matrix, the non-metallic inclusions and solid solution RE La can be separated by physicochemical separation methods, and all kinds of non-metallic inclusions can be distinguished. Based on the specific content relationship between inclusions and La, the solid solution and precipitation contents are evaluated in Figure 3. The result indicated that the plain carbon steel with different La content is composed of four types of inclusions: RE oxide, RE sulfur oxide, RE sulfide and solid solution RE. As the content of La addition increased from 67 ppm to 310 ppm, the content of La in inclusions increased from 66 ppm to 298 ppm, implying that the addition of La could modify the inclusions. The solution content of La was maximum when the addition of La was about 230 ppm.

**Figure 4:**
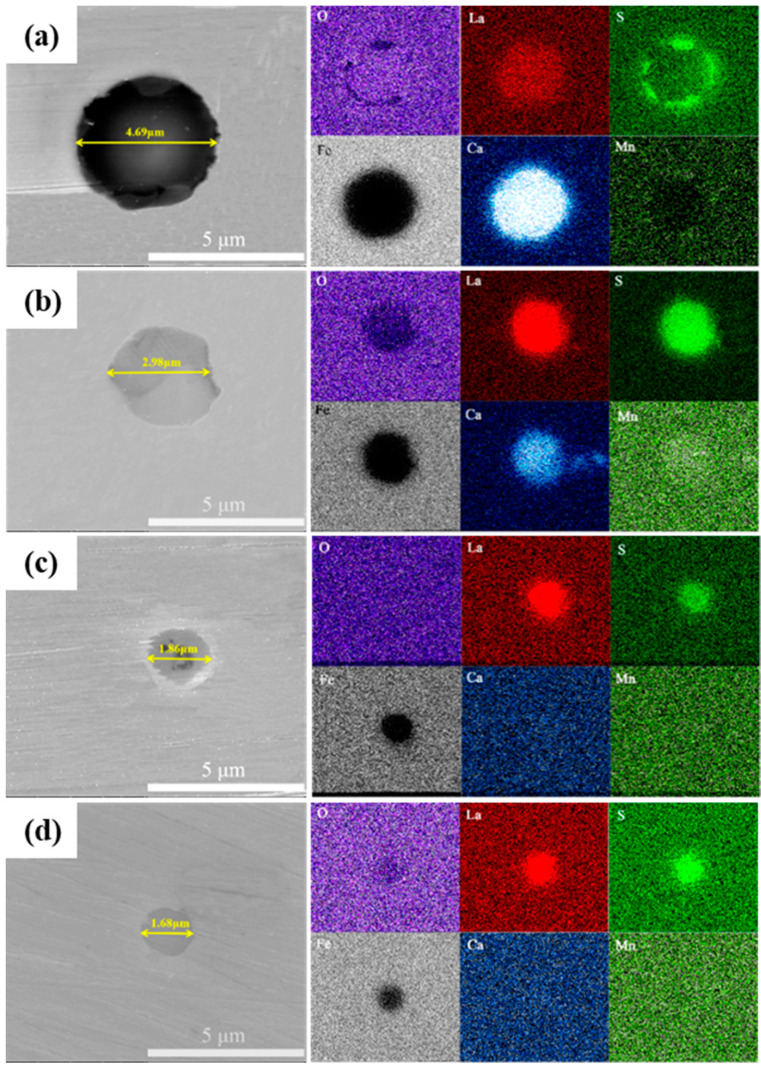
Inclusion EDS-mapping analysis by SEM: (**a**) 67La steel; (**b**) 110La steel; (**c**) 230La steel; (**d**) 310La steel.

Furthermore, the inclusions were analyzed by SEM with EDS mapping (Figure 4). As the content of La increased from 67 ppm to 310 ppm, the particle size of inclusion was refined from 4.69 μm to 1.68 μm, indicating that the modification effect of La content on inclusions is significant. 

When micro-alloyed by 67 ppm La, the big-sized inclusions were partially modified. For inclusion shape, the inclusions changed from irregular shape to spherical, and the size of the inclusions was reduced. The EDS-mapping result illustrated that the Ca element is enriched in this inclusion, verifying the presence of CaO. Furthermore, the content of Mn in the inclusion is reduced, implying that the original inclusion MnS has been modified. At the edge of this inclusion, it was mainly CaS inclusion under the presence of a high content of S.

As the content of La increased to 110 ppm, the inclusion size was about 2.98 μm, presenting as subspherical. Different from the inclusion in 67La steel, the distribution range of the Ca element was smaller than the La element, which is consistent with the distribution range of S. With the increase of La to 230 ppm, the inclusion size further decreased to 1.86 μm. The SEM-EDS mapping result showed that there was little Ca in this inclusion. With a further increase of La to 310 ppm, both the inclusion size was refined and the content of Ca decreased. These results indicate that RE La could significantly modify the inclusion.

**Figure 5:**
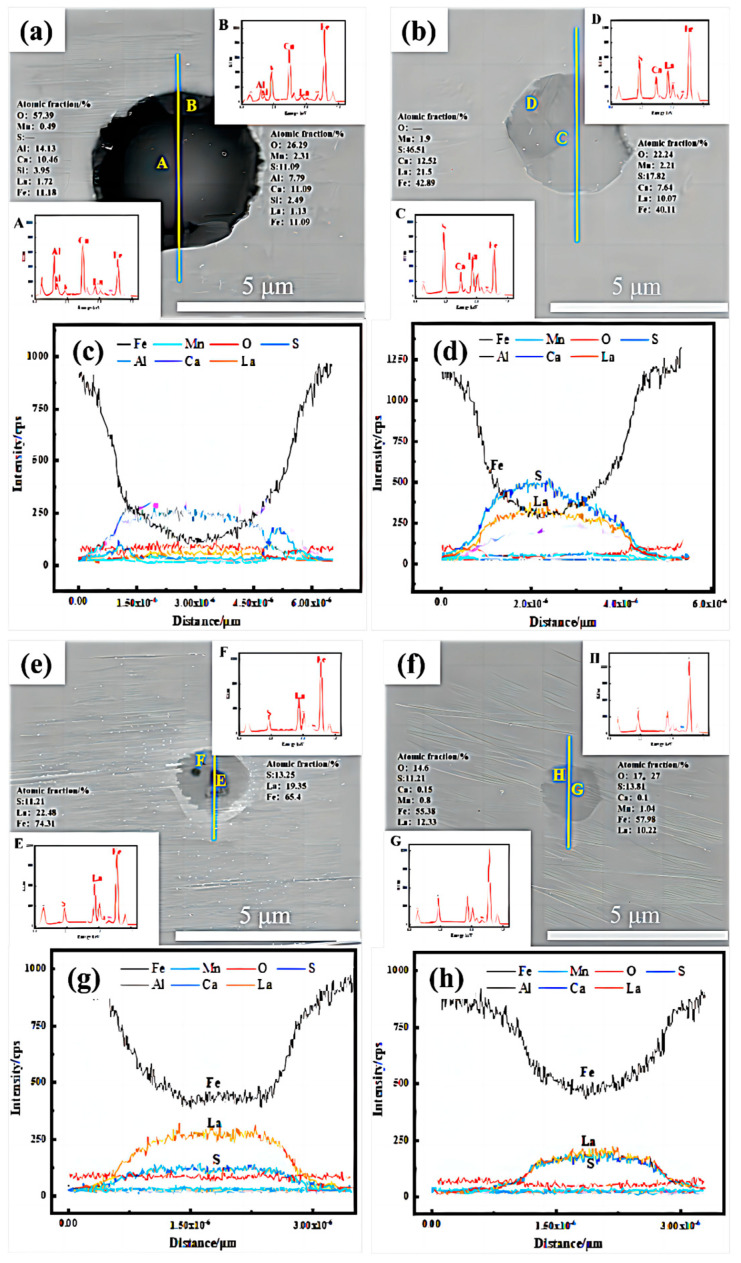
Inclusion EDS-line scanning analysis by SEM: (**a**,**c**) 67La steel; (**b**,**d**) 110La steel; (**e**,**g**) 230La steel; (**f**,**h**) 310La steel.

SEM-EDS line scanning results of inclusions are displayed in Figure 5. The results indicated that RE sulfide is mainly formed after the addition of La, which is caused by the strong ability of La to combine with S, O and other elements. In the inclusions with different La contents, the interior is first modified, and a low concentration of Mn and a high concentration of La can be found in the center of the inclusions. However, for inclusions modified by low-content La, Ca, Al and Si elements were found to replace Fe. With the increase of RE La, the composition and morphology of inclusions changed. The strong binding ability of RE with S and O is reflected, and the inclusion is mainly in the form of rare earth sulfide.

### 3.3. Corrosion Inhibition Rate

**Figure 6:**
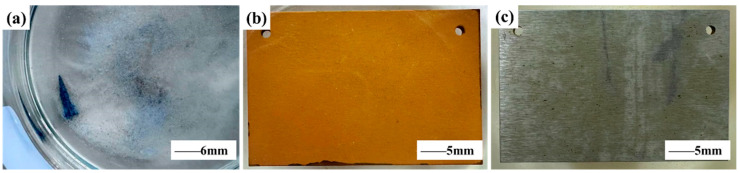
Dissolution and Corrosion Morphologies. (**a**) Dissolution morphology of pure RE La specimen in 0.01 mol/L NaHSO_3_ (pH = 3.2) solution. Macroscopic corrosion morphology of plain carbon steel after 2 days of immersion in different corrosive media: (**b**) 0.01 mol/L NaHSO_3_ solution, and (**c**) 0.01 mol/L NaHSO_3_ + 0.75 g/L RE La.

The pure RE La specimen was placed in a 0.01 mol/L NaHSO_3_ (pH = 3.2) solution (Figure 6). It was found that the pure La RE specimen could react and dissolve in the acidic NaHSO_3_ solution, releasing La^3^⁺ ions. Figure 6 shows the corrosion morphology of plain carbon steel after immersion in different corrosive media for 2 days. The results depicted that in the 0.01 mol/L NaHSO_3_ solution, the presence of La^3^⁺ ions could greatly reduce the corrosion of carbon steel. After the carbon steel was immersed in the 0.01 mol/L NaHSO_3_ solution for 2 days, a large area of a brownish-yellow rust layer was formed on the sample’s surface. Serious corrosion occurred on the surface of the carbon steel, and the coverage rate of the rust layer was 100%. However, after immersion in 0.01 mol/L NaHSO_3_ + 0.75 g/L RE La solution for 2 days, there was no obvious color change at the surface of the carbon steel, indicating the weak corrosion degree. The corrosion inhibition rate of carbon steel was close to 75% after 2 days of total immersion. The results of the total immersion corrosion test show that La^3^⁺ ions produced by the unstable hydrolysis of the pure La rare earth block have a remarkable corrosion inhibition effect.

### 3.4. Alternate Immersion Test

**Figure 7:**
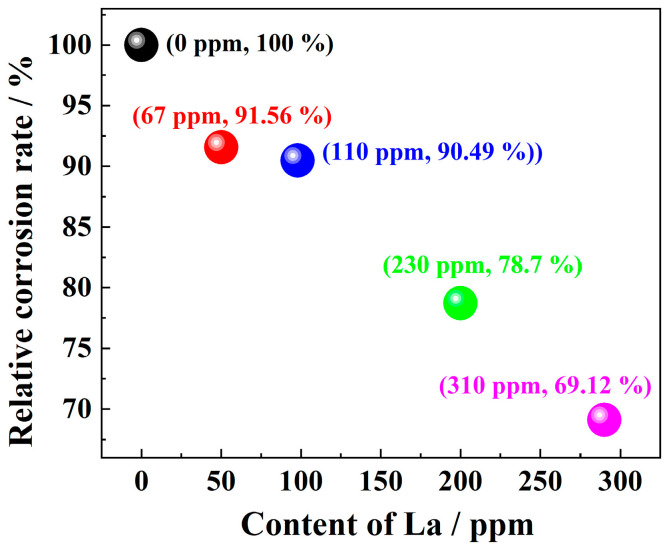
The relative corrosion rate of plain carbon steels micro-alloyed by different contents of La.

Figure 7 shows the relative corrosion rate of plain carbon steels with different La contents under simulated atmospheric corrosion conditions for 72 h. The results show that the trace RE La could significantly improve the corrosion resistance of plain carbon steel, and the corrosion inhibition performance was almost positively correlated with the total amount of RE La. The corrosion resistance of La-bearing steels has been continuously improved. La element could improve the corrosion resistance of steel by up to 30%, considering that the addition of RE La is less than 300 ppm, indicating that La has significantly improved the corrosion resistance of steel. When only 67~110 ppm of RE La was added, the relative corrosion rate of carbon steel was reduced by about 10%. It could be seen that the corrosion inhibition effect of RE La still exists even at low concentrations.

### 3.5. Rust Analysis

**Figure 8:**
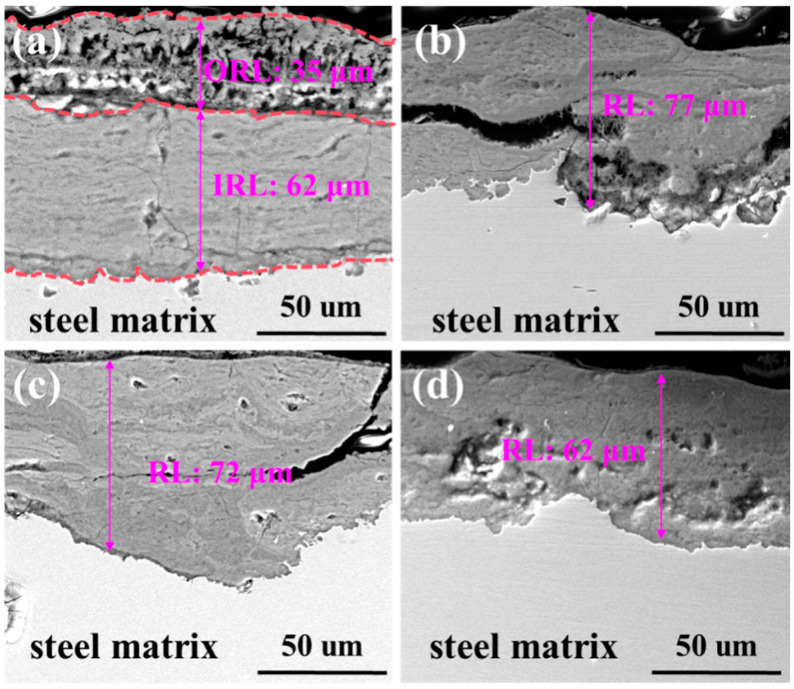
Cross-sectional morphology of La-bearing carbon steels with a 72-h rust layer: (**a**) 67 ppm; (**b**) 110 ppm; (**c**) 230 ppm; (**d**) 310 ppm.

The morphology of the rust layer on the surface of plain carbon steels with different La contents was observed after the alternate immersion accelerated corrosion tests for 72 h (Figure 8). With the increase of La content, the thickness of the rust layer gradually decreased from 97 μm to 62 μm, indicating that the degree of corrosion breakage was reduced. The rust layer of 67La steel was divided into two layers: the outer rust layer (ORL) and the inner rust layer (IRL). The ORL with 35 μm thickness had poor density and loose structure, and the IRL with 72 μm thickness was relatively dense, showing that the La-bearing steel has certain corrosion resistance. Micro-alloyed by 110 ppm La, the rust layer thickness decreased to 77 μm, while it became denser. Additionally, a large-size second crack appeared in the rust layer of 110La steel. For 230La steel, the rust layer thickness decreased to 72 μm, the dense of the rust layer increased, and there was also a large-size second crack. As the content of La increased to 310 ppm, the rust layer of the steel was densest, and no obvious cracks and voids were observed, implying uniform corrosion. It could be seen that the corrosion inhibition of La caused the formation of a stable protective rust layer on the steel surface. 

**Figure 9:**
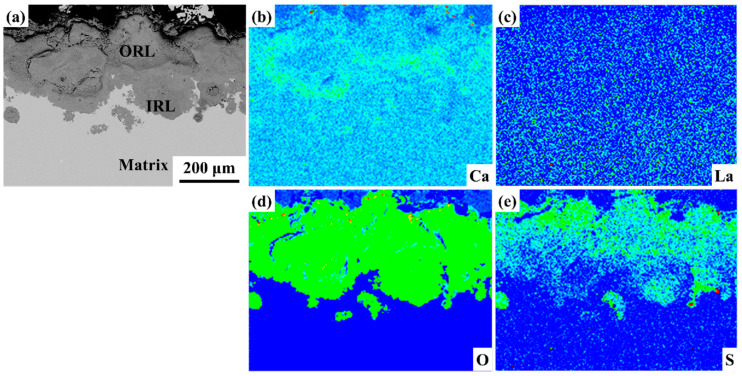
Electron probe microanalysis of rust structure of 230La steel: (**a**) SEM image micrograph. Mapping results of (**b**) Ca, (**c**) La, (**d**) O and (**e**) S.

The element distributions in rust by EPMA are shown in Figure 9. The outer rust layer and inner rust layer were observed. The EPMA result indicated that Ca is enriched in the inner rust layer, while La is mainly distributed in the inner rust layer and matrix. O element was approximately evenly distributed in the outer and inner rust layers. Differently, the S element is concentrated in the outer rust layer.

**Figure 10:**
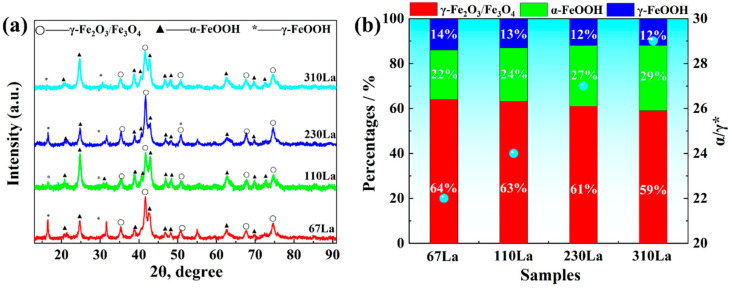
(**a**) XRD patterns of corrosion products after 72 h of alternate immersion accelerated corrosion test, and (**b**) Semi-quantitative analysis of corrosion products after 72 h of alternate immersion accelerated corrosion test.

The XRD pattern of corrosion products of plain carbon steel with different La contents in 0.01 mol/L NaHSO_3_ solution is shown in Figure 10a. The results indicate that the rust layer was mainly composed of α-FeOOH, γ-FeOOH and Fe_3_O_4_. With the increase of RE La content, the content of γ-FeOOH with higher electrochemical activity decreased, while the content of α-FeOOH with lower electrochemical activity increased, thereby forming a more dense and stable rust layer. Figure 10b shows the phase semi-quantitative analysis of corrosion products of plain carbon steel with different La contents after a 72 h alternate immersion accelerated corrosion test. As the content of La increased from 67 ppm to 310 ppm, the fraction of γ-Fe_2_O_3_/Fe_3_O_4_ decreased from 64% to 59%, and the fraction of γ-FeOOH also decreased from 14% to 12%, while the fraction of α-FeOOH increased from 22% to 29%. 

### 3.6. Potentiodynamic Polarization Tests

**Figure 11:**
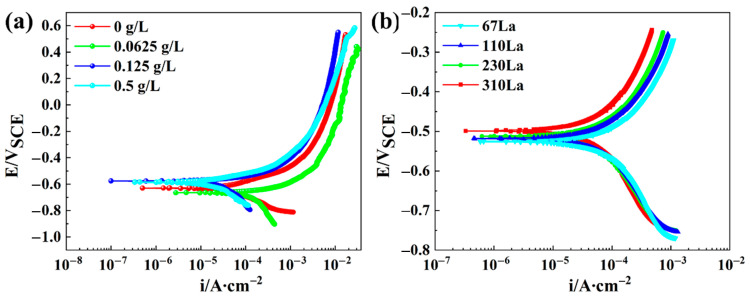
Polarization curves: (**a**) 0La plain carbon steel in 0.01 mol/L NaHSO_3_ solution with different mass concentrations of La^3+^; (**b**) Different La contents in 0.01 mol/L NaHSO_3_.

Electrochemical tests were performed on the plain carbon steels without La addition in different La-bearing solutions: 0 g/L, 0.0625 g/L, 0.125 g/L, and 0.5 g/L. The polarization curves are displayed in Figure 11, and the fitted result of corrosion current and potential are listed in Table 2. The initial corrosion potential and current for 0La steel in a single 0.01 mol/L NaHSO_3_ solution was −785 mV and 4.04 × 10^−4^ A/cm^2^, respectively. As the 0.0625 g/L LaCl_3_ was added in 0.01 mol/L NaHSO_3_ solution, the corrosion potential decreased to −840 mV and the corrosion current also decreased to 4.04 × 10^−4^ A/cm^2^. As the content of LaCl_3_ increased to 0.125 g/L, the corrosion potential increased to −732 mV, while the corrosion current decreased to 8.76 × 10^−5^ A/cm^2^. When the content of LaCl_3_ was 0.5 g/L, the corrosion potential decreased to −740 mV, while the corrosion current increased to 9.01 × 10^−5^ A/cm^2^. These results indicated that the La^3+^ ion could act as a corrosion inhibitor, reducing the corrosion rate.

### 3.7. Electrochemical Impedance Spectroscopy (EIS)

**Figure 12:**
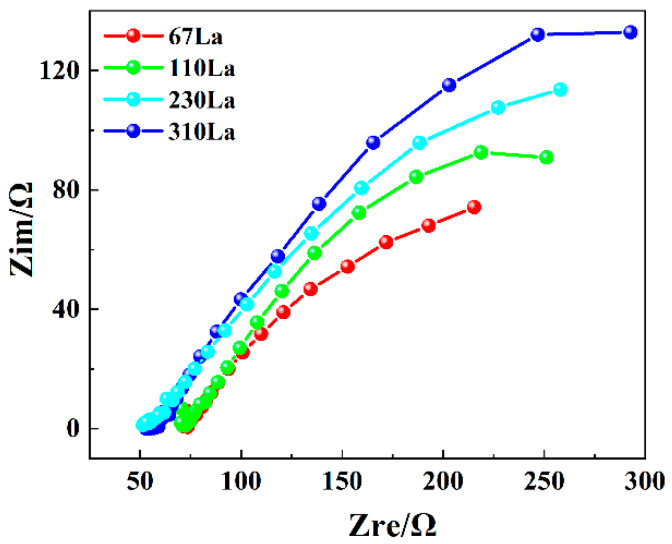
Nyquist curves of steel with different La contents in 0.01 mol/L NaHSO_3_ solution.

The Nyquist curves can show the electrical behavior between the rust layer and the electrolyte. In the Nyquist curves, the characteristics of the high-frequency region are related to the properties of the rust layer, reflecting the charge transfer process of the double layer. The characteristics of the low-frequency region are related to the electrode process at the rust layer/metal interface, reflecting the diffusion process of the electrolyte through the rust layer in Figure 12. Therefore, the Nyquist curves fully reflect the properties of the rust layer and the interaction between the electrolyte and the rust/metal interface.

EIS experiments on four kinds of La-bearing carbon steels show that their evolution characteristics were similar. In the high-frequency region, the Nyquist curves of La-bearing steels showed the characteristics of capacitive reactance arc and diffusion arc. With the increase of rare earth content, the rust layer on the surface of La-bearing plain carbon steel gradually increased and became more stable, thus resulting in the characteristics of a capacitive reactance arc. In addition, the arc radius of capacitive reactance also increased, which indicated that the stability of the rust layer on the surface of the La-bearing plain carbon steel is further improved.

## 4. Discussions

### 4.1. Microalloying Mechanism of La

The important roles of RE La element in steels can be summarized as purifying molten steel, modifying inclusions, and corrosion resistance [25]. The principle for purifying the liquid steel is located at the strong chemical affinity between RE and oxygen or sulfur [26], reducing the content of harmful elements. Therefore, the main existing forms of La in steel were the inclusion [27], intermetallic compound [28] and solid solution. Through the physicochemical separation of non-metallic inclusions and solid solution RE La, the types and quantities of inclusions were classified and counted. The content of solid solution RE La is the difference between the total addition of RE and the content in non-metallic inclusions. However, the intermetallic compound formed under the specific alloying elements, and the intermetallic compound was not found. The tested existing forms were inclusions and solid solutions in present La-bearing steels. The inclusions were sulfides, sulfur oxides, and oxides.

**Figure 13:**
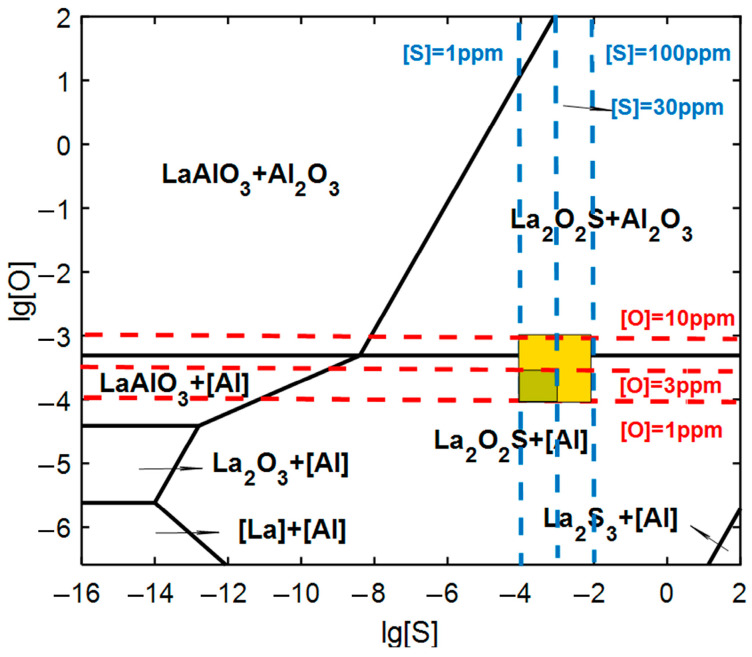
Relationship between sulfur content and activity oxygen in molten steel with the addition of lanthanum.

As the content of La increased, the size of oxide and sulfide inclusions decreased. The fraction of other nonmetallic inclusions also decreased. To solubilize RE in plain carbon steel, it is necessary to control the content of inclusions. RE_x_S inclusions are the main ones, and inclusions such as RE_x_O_y_, RE_2_O_2_S and ReAlO_3_ are not expected. The standard Gibbs free energy change for the formation of different inclusions is listed in Table 3. Based on the equation, the inclusion types varying with the content of [O] and [S] were displayed in Figure 13.

For plain carbon steel, the actual refining process needs to control the content of oxygen and sulfur. The total oxygen content is controlled in 1–3 ppm and the content of sulfur is controlled in the range of 10~20 ppm. This allows the formation of the La_2_O_2_S phase and avoids the formation of the La_2_S_3_ phase. This could reduce the content of inclusions. Therefore, precise control of oxygen and sulfur content is the key, which will help to obtain higher quality solid solution materials after the addition of lanthanum in rare earth steel.

According to the SEM-EDS results, when the content of rare earth La was 67 ppm, there was still Ca enrichment in the inclusion. Besides, the inclusion still had calcium oxide, but the internal Mn content was reduced, indicating that the original inclusion of MnS has been modified. With the increase of RE La, the enrichment degree of calcium gradually decreased, indicating the strong binding force of La and O in liquid steel. When rare earth La was added to 230 ppm, the oxides in the steel were mainly RE sulfurs and oxides, and the inclusions were modified more thoroughly. Because the O content in 310La steel is relatively small, after adding 310 ppm La, the inclusions are mainly RE sulfides.

Under the total content of La of 230 ppm, the solution content of La in steel reached 66 ppm, which was highest in present plain carbon steels. The RE La in a solid solution state plays a great role in γ→α transformation, mechanical properties and corrosion resistance.

### 4.2. Effect of La on Microstructure, Strength, and Toughness

As the content of La increased, the content of pearlite decreased, while the content of acicular ferrite increased, resulting in the refined microstructure. The mechanism of RE on refining microstructure was as follows: (a) The effective RE inclusions could act as the heterogeneous nucleation nucleus of acicular ferrite by the low mismatch theory [32,33]; (b) La atoms, with big-size atomic radius, could play the role of solid solution drag effect [34,35], restricting the diffusion-type transformation. The present microstructure types were all diffusion-type matrix transformations with the migration of C atoms. Thermodynamically, both the polygonal ferrite and acicular ferrite would occur under the coiling temperature of 500 °C. Both the polygonal ferrite and acicular ferrite transformations started from prior austenite grain boundaries [36]. Meanwhile, C atoms diffused from ferrite to undercooled austenite during γ/α transformation migration. The C-rich austenite promoted the formation of granular bainite and pearlite. C atoms mainly concentrated in martensite–austenite constitute in the former, while they were in cementite in the pearlite. Pined by the La atoms in the solution state, the interface migrating velocity decreased, refining the microstructure.

As the content of La increased, the addition of trace microalloying elements could improve the trade-off of strength and impact energy. When the content of 67 ppm La was added, the tensile strength and yield strength increased by 47 and 61 MPa, respectively. The increase in strength was attributed to the refined microstructure, where the fraction of granular bainite was highest. Although the higher content of La refined the microstructure, the strength appeared slight decrease, due to the decrease of granular bainite.

Within the La addition of 230 ppm, the room-temperature impact energy was optimized, rising about 22.6% compared to 0La steel. Once the content of La increased to 310 ppm, the impact energy decreased to 53 J. Although the fraction of pearlite was the lowest and the inclusion size was the smallest, the amount of inclusion increased. Warren, et al. [37] found that the La addition of 470 ppm damaged the impact toughness of AF1410 steel (213 J to 127 J), while the addition of 120 ppm La significantly increased the impact energy from 61 J to 88 J. Therefore, the total addition content of La was appropriate, avoiding too many particles damaging comprehensive properties.

### 4.3. Effect of La Element on Corrosion Resistance

To quantitatively evaluate the corrosion inhibition efficiency of the La element in plain carbon steel, the conception of an effective RE La formula was introduced. Compared with RE La sulfides and RE La sulfur oxides, RE La oxides are extremely difficult to dissolve or decompose because of their more compact and ordered crystal structure and high chemical stability.

Therefore, in the effective RE formula, the coefficient of RE oxides was set to 0. The RE sulfur oxides and RE sulfides in plain carbon steels are generally La_2_O_2_S and La_2_S_3_. To simulate the industrial atmospheric environment, 0.01 mol/L NaHSO_3_ solution was used in the alternate immersion test box. As a reductant, sodium bisulfite can react with RE sulfide and RE sulfur oxide to accelerate the release of La^3+^ ions. The crystal structure of RE sulfide is relatively loose and its bond force is weak. Therefore, compared with rare earth sulfur oxides, the solubility of rare earth sulfide in sodium bisulfite solution is higher. Due to the higher electronegativity of oxygen atoms, RE sulfur oxides form stronger chemical bonds with RE elements, resulting in a more stable crystal structure, so the solubility of RE sulfur oxides is low. According to the different solubility and corresponding action mechanisms of RE sulfide and RE sulfur oxide, the coefficient of RE sulfide was set to 0.4, and the coefficient of RE sulfur oxide was set to 0.2. Through this formula, the influence of different La contents on the corrosion inhibition rate of carbon steel can be more accurately evaluated. The specific effective RE La formula is as follows:

(4) $$V_{\mathrm{La}}=0.4\times V_{\mathrm{La}\text{-}S}+0.2\times V_{\mathrm{La}\text{-}O\text{-}S}+V_{\mathrm{La}\text{-}M}$$

where $V_{\mathrm{La}}$ is the effective RE La content, $V_{\mathrm{La}\text{-}S}$ is the RE La in sulfide, $V_{\mathrm{La}\text{-}O\text{-}S}$ is the RE La in sulfide oxides, and $V_{\mathrm{La}\text{-}M}$ is the RE La in a solid solution state.

**Figure 14:**
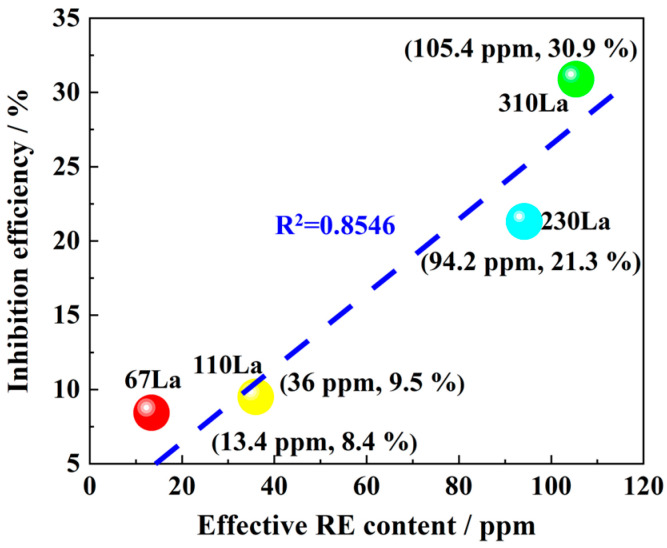
Corrosion inhibition rate and effective RE content for La-bearing plain carbon steel.

Figure 14 shows the corresponding relationship between effective RE content and corrosion inhibition rate obtained by substituting plain carbon steel with different additions of La. The result indicates that there is an almost linear positive correlation between the effective RE La content and the corrosion inhibition rate of plain carbon steel. When the effective RE La content increased from 94.2 ppm to 105.4 ppm, the corrosion inhibition rate increased from 21.3% to 30.9%. This was mainly because the RE La^3+^ ion, as a corrosion inhibitor, effectively inhibited the corrosion process of plain carbon steel. The presence of La^3+^ ions would also cause the local pH value on the surface of carbon steel to rise, hindering the dissolution and oxidation process of metal ions.

For the high-frequency band, the capacitive arc reactance has a charge and discharge capacity similar to that of capacitors, so its capacitance-related ability is relatively strong, but some non-conductive substances in the rust layer will affect the transmission of electrons, so the resistance value could not be ignored. After the impressed current is charged first, the electron transfer process between the corrosion product and the corrosion solution is reflected when the current passes through the rust layer, thus reflecting the thickness of the rust layer and the related properties of the corrosion product. At this stage, the rust layer resistance Rr and the rust layer capacitance C1 jointly control the equivalent circuit.

In the low-frequency band, the interface between the matrix and the corrosion solution forms a double layer of capacitive arc resistance, which also has the function of charge and discharge. Due to the long reaction time, many electrons will accumulate on the substrate surface, resulting in a concentration difference. At this stage, concentration polarization resistance and electrochemical impedance work together,

The corrosion reaction anode region of carbon steel containing La would undergo the following chemical reactions [15]:

NaHSO_3_ = Na^+^ + H^+^ + SO_3_^2−^ (5)

2SO_3_^2−^ + O_2_ = 2SO_4_^2−^ (6)

Fe − 2e^−^ = Fe^2+^ (7)

Fe^2+^ + SO_4_^2−^ = FeSO_4_ (8)

4FeSO_4_ + O_2_ + 6H_2_O = 4FeOOH + 4H_2_SO_4_ (9)

2H_2_SO_4_ + 2Fe + O_2_ = 2FeSO_4_ + 2H_2_O (10)

La − 3e^−^ = La^3+^ (11)

La_2_S_3_ + 6H^+^ = 2La^3+^ + 3H_2_S (12)

La_2_O_2_S + 6H^+^ = 2La^3+^ + H_2_S + 2H_2_O (13)

The cathode part will undergo the following reaction:

2H^+^ + 2e^−^ = H_2_ (14)

3FeOOH + H^+^ + e^−^ = Fe_3_O_4_ + 2H_2_O (15)

2H_2_O + O_2_ + 4e^−^ = 4OH^−^ (16)

The above reactions in the cathode will increase the concentration of OH^−^ ions and the pH value in the micro-cathode region, creating conditions for the deposition of RE ions. The RE ions dissolved in the corrosive medium will undergo the following precipitation reactions to slow down the corrosion process:

La^3+^ + 3OH^−^ = La(OH)_3_↓ (17)

2La(OH)_3_ = La_2_O_3_ + 3H_2_O (18)

With the increase of La content, the density of the rust layer was continuously improved, which can effectively reduce the interaction between carbon steel and oxygen and water in the environment, thus reducing the corrosion rate. 

With the increase of RE La content, the proportion of corrosion product Fe_3_O_4_ in tested steel gradually decreases, showing that the La element could inhibit the formation of Fe_3_O_4_. As a conductive phase, Fe_3_O_4_ is likely to cause cracking of the dense rust layer, thus reducing the corrosion resistance of the matrix. As an insulator, α-FeOOH could play a certain hindering role in the transfer of charge, so that the electrochemical reaction was difficult to occur, indicating a good protective effect on the steel matrix. 

## 5. Conclusions

The main conclusions were displayed as follows:

(1) The microalloying of La element modified the inclusion from mixed Al_2_O_3_·CaO·CaS inclusion to LaS, LaO and La_3_(SO)_2_ inclusion. Although the total content of inclusion increased with the content of La, the particle size decreased from 4.69 μm of 67La steel to 1.68 μm of 310La steel. The maximum La content in the solid solution state reached 66 ppm in 230La steel, while the content of La in the solid solution state decreased to only 12 ppm.
(2) As the content of La increased, the microstructure was refined. The fraction of pearlite decreased, and the content of acicular ferrite increased. The modified inclusions and microstructure jointly improved the strength and toughness within the La addition of 230 ppm for Q355 steel. The yield strength of 419 MPa, tensile strength of 569 MPa, and impact energy of 76 J were obtained.
(3) La element plays a significant role in corrosion inhibition. The addition of La significantly reduces the cracks and holes in the rust layer and reduces the thickness of the rust layer. The release of La^3+^ ions promoted the formation of a dense and continuous protective rust layer, which effectively inhibited the corrosion process. The effective proposed RE formula accurately quantified the corrosion inhibition efficiency of La elements, and revealed the positive correlation between the effective rare earth content and the corrosion inhibition rate.

## Figures and Tables

**Table 1:** Chemical compositions of the tested steels (weight percentage, wt.%).

Samples	C	Si	Mn	P	S	O	N	La
0La	0.16	0.29	1.24	0.012	0.0120	0.0007	0.020	0
67La	0.16	0.28	1.36	0.0068	0.002	0.0028	/	0.0067
110La	0.16	0.28	1.33	0.007	0.0081	0.0017	0.0035	0.011
230La	0.16	0.28	1.33	0.0066	0.0032	0.0026	/	0.023
310La	0.16	0.28	1.34	0.0069	0.0076	0.0010	0.0058	0.031

**Table 2:** Fitting results of polarization curves of plain carbon steel in 0.01 mol/L NaHSO_3_ solution with different mass concentrations of La^3+^.

Sample	Ecorr/mV	icorr/A·cm^−2^
0La–0 g/L	−785.0	4.04 × 10^−4^
0La–0.0625 g/L	−840.0	3.22 × 10^−4^
0La–0.125 g/L	−732.0	8.76 × 10^−5^
0La–0.5 g/L	−740.0	9.01 × 10^−5^
67La–0 g/L	−525.72	2.52 × 10^−5^
110La–0 g/L	−520.61	1.91 × 10^−5^
230La–0 g/L	−513.03	1.15 × 10^−5^
310La–0 g/L	−498.07	6.65 × 10^−6^

**Table 3:** The standard Gibbs free energy for inclusions [29,30,31].

Reaction Equation	Δ*G^θ^* = A + B × *T* (J/mol)	
	A	B
[Mn] + [S] = MnS(s)	−158,365	93.966
2[Al] + 3[O] = Al_2_O_3_(s)	−122,500	393.8
2[La] + [O] = La_2_O_3_(s)	−1,443,880	337
2[La] + 2[O] + [S] = La_2_O_2_S(s)	−1,341,200	301
[La] + [S] = LaS(s)	−445,180	141.5
[La] + [Al] + 3[O] = LaAlO_3_(s)	−801,616	28.9

## Data Availability

The data presented in this study are available on request from the corresponding author.
